# Changes in the hemolytic activity of *Candida* species by common electrolytes

**DOI:** 10.1186/s12866-015-0504-7

**Published:** 2015-08-22

**Authors:** Lei Wan, Gang Luo, Haibin Lu, Dongying Xuan, Hong Cao, Jincai Zhang

**Affiliations:** Department of Microbiology, School of Public Health and Tropical Medicine, Southern Medical University, Guangzhou, China; Department of Periodontology, Guangdong Provincial Stomatological Hospital, Southern Medical University, Guangzhou, China; Key Laboratory of Oral Medicine,Guangzhou Institute of Oral Disease,Stomatology Hospital of Guangzhou Medical University, Guangzhou, 510140 China

## Abstract

**Background:**

Hemolysins are crucial virulence factors which help pathogens to survive and persist in the host. This study investigated whether common electrolytes will affect the hemolysins of *Candida* species. The hemolysins from 25 *Candida* isolates were investigated using a plate specially designed for *Candida* species in the presence of three electrolytes, CaCl_2_, NaCl and KCl, at different concentrations. The hemolytic activity was determined after 48 h and the hemolytic index was calculated.

**Results:**

All three electrolytes caused a decrease in the hemolytic activity. Significant differences existed between varying concentrations of NaCl, while no significant differences existed for the CaCl_2_ and KCl groups. Additionally, the peripheral hemolytic index was highly correlated with the hemolytic index (*r* = 0.656, *p* < 0.001).

**Conclusions:**

Our findings indicate that electrolytes reduce hemolysis by *Candida* species and a correlation exists between the peripheral hemolytic index and the translucent hemolytic index.

## Background

Pathogenic fungi have become an increasing risk factor for people suffering from systemic disease and patients with impaired immune systems. Numerous fungi have been described, but only a small group, including *Candida* species, is pathogenic. *Candida* species can cause superficial mucosal infections and deadly systemic disease. In recent years, the number of *Candida* infections has increased dramatically and many have resulted in mortality [1–4].

Therefore, a better understanding of *Candida* species pathogenicity will undoubtedly be beneficial for the treatment of *Candida* infections. Although the epidemiology of *Candida* species has been thoroughly studied, the virulence is not well understood. Virulence factors, such as adherence, extracellular hydrolase production, hemolysis, phenotypic switching, and filamentation may all influence the pathogenesis of *Candida* species. A variety of virulence factors are expressed by *Candida albicans* for the adaptation to specific anatomical sites [5]. As one of the most important virulence factors of *C. albicans*, secreted aspartate proteinases (Saps) have been studied fully [6], while its hemolytic activity is not well understood. Hemolytic activity is a potential virulence factor that helps to disseminate candidiasis and facilitate hyphal invasion [7]. The hemolytic activity of *Candida* is enhanced by growth on glucose-enriched blood agar, as first described by Manns et al. [8]. *Candida albicans* uses hemolysins to degrade hemoglobin and obtain elemental iron. Therefore, hemolysins are crucial virulence factors that help pathogens to survive and persist [8–10], but for *Candida* isolates, the features of hemolysins are poorly characterized [11].

A limited number of studies have explored the influence of specific ions on the hemolysins of *Candida* isolates. N. Yigit and Aktas [12] compared the use of different blood media on the hemolysins of *Candida* isolates and found that sheep blood Sabouraud dextrose agar is the most suitable for the study of the beta-hemolytic activity of *Candida* isolates, and Linares et al. [13] observed that CaCl_2_ affected the hemolytic activity. When 2.5 % CaCl_2_ was added to Sabouraud glucose agar supplemented with sheep blood, the hemolytic activity of *C. dubliniensis* was reduced and that of the *C. albicans* strains was stimulated. However, in the absence of 2.5 % CaCl_2_, the hemolytic activities of *C. albicans* and *C. dubliniensis* were not different.

Despite these previous studies, the effect of specific electrolytes on the hemolysins of *Candida* strains remains unclear. In particular, the relationship between common electrolytes and the hemolysins of *Candida* strains has not been explored. Although hemolysis may be effective at promoting the pathogenesis of *Candida* strains, additional studies of potential hemolytic factors are needed. This study was designed to test the influence of specific electrolytes on the hemolysins of *Candida* strains.

## Methods

### *Candida* species

Sixteen strains of *C. glabrata*, four *C. albicans* and one *C. tropicalis* recovered from clinical specimens of different patients at the Guangzhou Eighth People’s Hospital and preserved in the laboratory were included in the study. Additionally, a single strain each of *C. albicans* (ATCC 90028) (American Type Culture Collection, Manassas, VA, USA), *C. glabrata* (ATCC 90030), *C. krusei* (ATCC 6258) and *C. tropicalis* (ATCC 13803) were included for data comparison purposes. The API 20C Aux Identification Kit (Bio Merieux SA, Lyon, France) and the “germ tube test” were used to identity all the isolates. Cultures were stored at −79 °C. Once recovered from the patients, they were kept on Sabouraud dextrose agar (Guangzhou Detgerm Microbiology Technology Co., Guangzhou, China), and stored at 4 °C. The identification of each isolate was performed separately to guarantee their purities.

All the clinical isolates used in this study, which were not traceable to the donors, have been used previously and details of their isolation have been described [10, 14]. The hemolytic activity of every strain used in this study was determined twice in the absence of added electrolytes. The three electrolytes, CaCl_2_, NaCl and KCl (Aladdin Industrial Corporation, Shanghai, China), were added during the preparation of the plates.

### Preparation of plates with various electrolytes at different concentrations

The control medium was prepared by adding 7 mL of fresh sheep blood (Hemostat, Dixon, CA, USA) to 100 mL of Sabouraud dextrose agar containing 3 % glucose (final concentration, wt/vol; Guangzhou Detgerm Microbiology Technology Co., Guangzhou, China). The pH was 5.6 ± 0.2. NaCl or KCl was added to the above medium at concentrations of 1, 2.5 or 5 % (wt/vol), while CaCl_2_ was added at concentrations of 0.5, 1 or 2.5 % (wt/vol).

### Quantification of hemolytic activity

The method of Luo et al. [14] was used to assess hemolytic activity. Isolates were cultured in Sabouraud dextrose agar at 37 °C for 24 h. Then, the cultures were collected and washed with sterile saline and a yeast suspension was prepared from an inoculum of 1 × 10^8^ cells/mL by hemocytometric counts. Ten microliters was spotted on each plate. Plates were cultured at 37 °C in a 5 % CO_2_ atm for 48 h. A translucent halo around the inoculum indicated the presence of hemolytic activity. The ratio calculated by dividing the total diameter of the colony plus the translucent halo by the diameter of the colony was defined as the hemolytic index (Hi), which represented the intensity of hemolysin production. Moreover, a dark ring was observed at the periphery of the distinctive translucent halo on every plate. The ratio calculated by dividing the diameter of the peripheral dark ring, the translucent halo and the colony by the diameter of the colony was defined as the peripheral hemolytic index (Hp), which represented the intensity of the peripheral hemolysin production.

The relative change of hemolytic activity for a specific electrolyte at a specific concentration compared with the control group was calculated as follows: the relative change of the test culture hemolytic index = (test culture hemolytic index − hemolytic index of the control culture)/hemolytic index of the control culture.

### Statistical analysis

All experiments were performed in duplicate. All data were assessed for homogeneity of variance using the Levene index and expressed as the mean ± standard deviation. Hemolytic activities in the presence of a specific electrolyte at different concentrations were analyzed by the repeated measures test. A one-way analysis of variance (ANOVA) was used to assess the degree of change in hemolytic activity for each electrolyte at different concentrations. If there was heterogeneity of variance, the rank-sum test was used instead of one-way ANOVA. Bivariate correlation analysis was applied to calculate the relationship between Hi and Hp. All statistical analyses were carried out using SPSS 13.0 (SPSS Inc., Chicago, IL, USA) and *p* < 0.05 was considered to be statistically significant, while *p* < 0.01 was considered highly statistically significant.

## Results

Three time points, 24, 48, and 72 h, were selected for analysis. At 24 h post-inoculation, only partial hemolysis was observed. At 72 h post-inoculation, the zone of hemolysis in some plates became opaque. This phenomenon became more common after prolonged incubation. Therefore, the 48-h time point was chosen for comparison, which was consistent with previous studies [12, 13, 15]. All the clinical strains and the standard strains showed hemolytic activity in the presence of added electrolytes under all tested conditions.

### Differences in the hemolytic activity of each electrolyte at different concentrations

#### Effect of calcium chloride on the hemolytic activity of *Candida* isolates

**Fig. 1:**
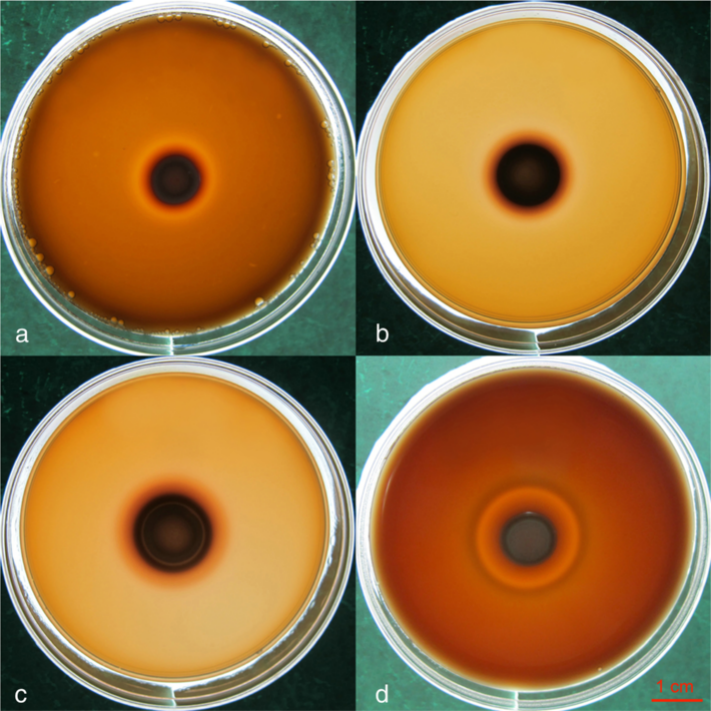
Hemolysis caused by *C. tropicalis* (ATCC 13803) on plates containing different concentrations of CaCl_2_. **a** 0.5 % CaCl_2_, **b** 1 % CaCl_2_, **c** 2.5 % CaCl_2_, and **d** control

To determine whether CaCl_2_ will affect the hemolytic activity of *Candida* isolates, we used plates supplemented with CaCl_2_ at 0.5, 1, or 2.5 % (wt/vol). Any decrease in the observed hemolytic activity was compared with the control plates. Comparison of the hemolytic indices among the groups treated with 0.5 % CaCl_2_ (Hi = 2.247 ± 0.079), 1 % CaCl_2_ (Hi = 2.013 ± 0.092), 2.5 % CaCl_2_ (Hi = 2.150 ± 0.066) and the control (Hi = 2.749 ± 0.103) reached statistical significance (*p* < 0.001). A typical result is shown in Fig. 1.

#### Effect of sodium chloride on the hemolytic activity of *Candida* isolates

**Fig. 2:**
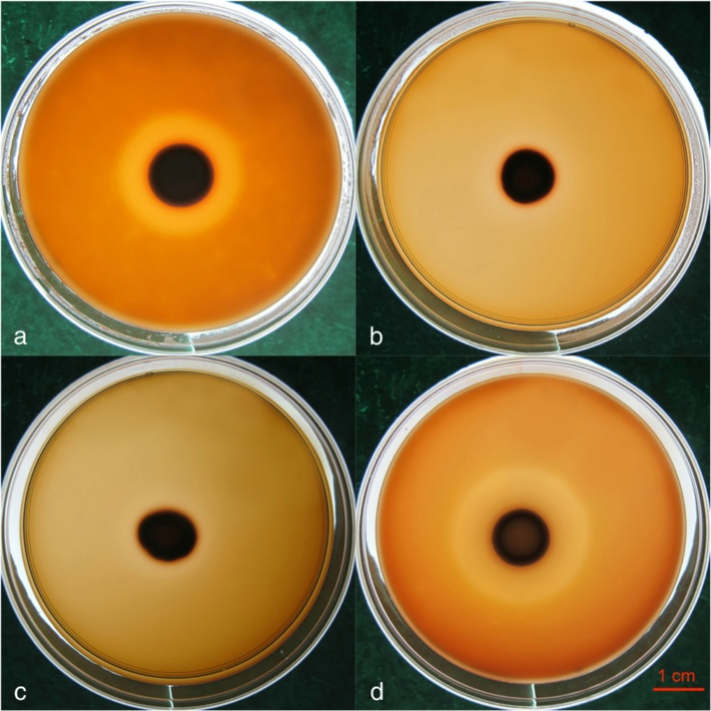
Hemolysis caused by a *C. glabrata* clinical strain on plates containing different concentrations of NaCl. **a** 1 % NaCl, **b** 2.5 % NaCl, **c** 5 % NaCl, and **d** control

To determine whether NaCl will affect the hemolytic activity of *Candida* isolates, plates including NaCl at 1, 2.5 or 5 % (wt/vol) were used. Any decrease in the observed hemolytic activity was compared with the control plates. Comparison of the hemolytic indices among the groups treated with 1 % NaCl (Hi = 2.158 ± 0.078), 2.5 % NaCl (Hi = 1.724 ± 0.077), 5 % NaCl (Hi = 1.746 ± 0.102) and the control (Hi = 2.839 ± 0.156) reached statistical significance (*p* < 0.001). A typical result is shown in Fig. 2.

#### Effect of potassium chloride on the hemolytic activity of *Candida* isolates

**Fig. 3:**
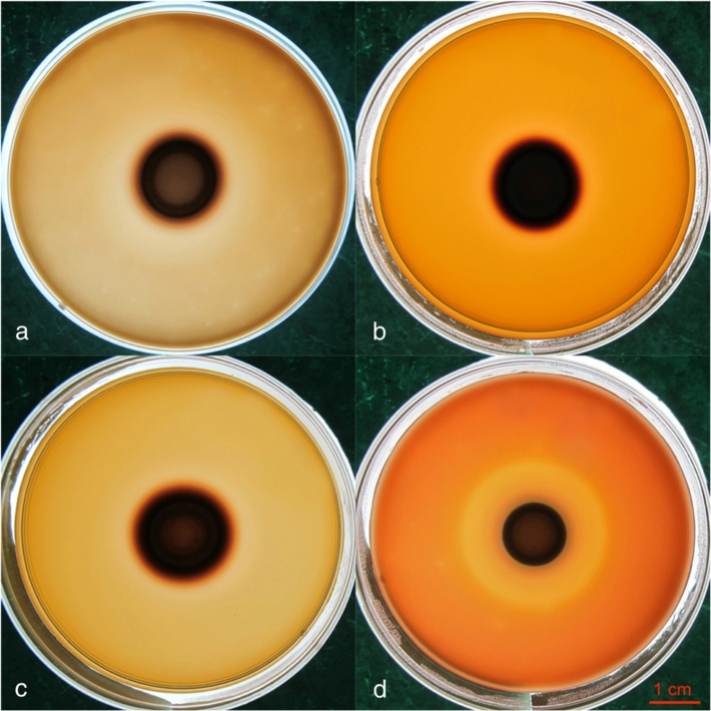
Hemolysis caused by a *C. albicans* clinical strain on plates containing different concentrations of KCl. **a** 1 % KCl, **b** 2.5 % KCl, **c** 5 % KCl, and **d** control

To determine whether KCl will affect the hemolytic activity of *Candida* isolates, plates containing KCl at 1, 2.5 or 5 % (wt/vol) were used. Any decrease in the observed hemolytic activity observed was compared with the control plates. Comparison of the hemolytic indices among the groups treated with 1 % KCl (Hi = 1.867 ± 0.077), 2.5 % KCl (Hi = 1.642 ± 0.079), 5 % KCl (Hi = 1.707 ± 0.089) and the control (Hi = 2.839 ± 0.156) reached statistical significance (*p* < 0.001). A typical result is shown in Fig. 3.

### Relative change of hemolytic activity in response to different concentrations of specific electrolytes

The relative changes in hemolytic activity for all three electrolytes at different concentrations were calculated according to the method described above. Significant differences existed between different concentrations for the NaCl group (*F* = 13.967, *p* < 0.001), while no significant differences existed between the different concentrations for the CaCl_2_ group (*F* = 2.727, *p* = 0.074). The Levene index of the KCl group was 0.001; therefore, the rank-sum test was used. The result of the KCl group was *χ*^2^ = 3.734, *p* = 0.155. For multiple comparisons among different concentrations, in accordance with a significance level of α = 0.05/3, no significant difference existed in the KCl group. The specific values are shown in Table 1.

**Table 1:** Relative change of hemolytic activity in response to different concentrations of specific electrolytes

	Levene index	Analysis of variance		Pairwise comparisons		
	*p*	F	*p*	A vs. B	A vs. C	B vs. C
				*p*	*p*	*p*
CaCl_2_	0.438	2.727	0.074	0.082	1.000	0.319
NaCl	0. 354	13.967	<0.001	< 0.001	< 0.001	1.000
KCl	0.001	3.642	0.033	0.040	0.145	1.000

A, B, and C each represent the concentration of an electrolyte and are arranged from lowest to highest. In the CaCl_2_ group, A represents 0.5 % CaCl_2_, B represents 1 % CaCl_2_ and C represents 2.5 % CaCl_2_, while in the NaCl and KCl groups, A represents 1 %, B represents 2.5 % and C represents 5 % of each respective electrolyte

### Relationship between Hp and Hi

After integrating all the data from the CaCl_2_ group, the NaCl group and the KCl group, the coefficient for the correlation between the peripheral hemolytic index and the hemolytic index was 0.656 (*p* < 0.001).

## Discussion

A previous study showed that the hemolytic activity of *C. dubliniensis* and *C. albicans* was affected by 2.5 % CaCl_2_ [13]. Therefore, we selected bracketing concentrations of 1, 2.5, and 5 % for studying the electrolytes NaCl and KCl. Because the addition of 5 % CaCl_2_ to the agar prevented the agar from solidifying, the concentrations chosen for the CaCl_2_ group were 0.5, 1, and 2.5 %.

Many factors can influence hemolytic activity, such as temperature [16], glucose [14], certain ions, including Fe^3+^ [17], certain compounds (ethanol, *n*-butanol, or *n*-pentanol vapor) [18]^,^ or the species that is the source of the blood [12]. Franca et al. [19] analyzed the hemolytic activities of *Candida* strains that were obtained from the same site and showed that *C. tropicalis* blood isolates had significantly higher activity. However, when they compared the hemolytic activities of *Candida* strains from different sites, the results showed that the hemolytic activity of *C. parapsilosis* isolates from tracheal secretions was higher. In addition, different batches of media, the thickness of the media, and the vitality of the strain can all affect the hemolytic activity of *Candida* species. However, some factors have no impact on the hemolytic activity. Favero [15] indicated that incubation in a normal environment or increased CO_2_ did not have any effect on hemolysis by *C. tropicalis*, nor was the hemolytic activity of *C. tropicalis* suppressed by heat treatment (100 °C) or by supplementation with pepstatin A. However, for beta-hemolytic *Streptococci*, an increased CO_2_ atm (5–10 %) promoted hemolysis [20]. Thus, an increased CO_2_ environment may also influence the hemolytic activity of *Candida* in a distinct manner [15].

Before we tested the effects of electrolytes on *Candida* species hemolysis, the hemolytic activity of every strain was evaluated twice in the absence of added electrolytes. All the strains showed hemolytic activity. After adding the electrolytes, every strain still showed positive hemolysis. However, our results suggested that when grown in CaCl_2_, NaCl, or KCl, the hemolytic activity of *Candida* isolates was reduced. In patients, the levels of these electrolytes depend on a myriad of factors. The relative balance of electrolytes is important to the health and function of the whole body, and electrolyte disorders are common in cardiovascular patients [21], in older community subjects [22], and in diabetic outpatients, even if their renal function is normal [23]. Additionally, patients with malignancies commonly experience serum electrolyte abnormalities, including hyponatremia, hypokalemia, hyperkalemia, hypophosphatemia, and hypercalcemia [24].

Electrolyte disorders are complicated to analyze in vivo, and it is impractical to simulate their complexity in vitro. In local environments, such as oral mucosa or vaginal mucosa, exogenous substances, such as food, drugs and rinses, can influence the electrolyte balances. The use of toothpaste containing calcium bicarbonate may be helpful for the prevention of oral candidiasis. Other strategies such as rinses containing certain electrolytes may be useful for the prevention of vaginal candidiasis. Whether an increase in the level of a specific electrolyte in a local environment will have an impact on the hemolytic activity of *Candida* isolates is currently unknown. Our results show that hemolytic activity in the presence of the three selected electrolytes was decreased compared with the control group. The relative change in hemolytic activity for all three electrolytes at different concentrations was also determined. No significant differences existed in the hemolytic activity of *Candida* between different concentrations of CaCl_2_ and KCl. However, significant differences were evident between the relative hemolytic activities in the presence of 1 % NaCl and 2.5 % NaCl, and 1 % NaCl and 5 % NaCl. A concentration of 1 % NaCl is slightly higher than the normal blood concentration (0.9 % NaCl) indicating that the hemolytic activity of *Candida* species under conditions of hypernatremia is decreased, which may be useful for the clinical management of patients with electrolyte disturbances.

To date, there are few studies of ions and their influence on hemolytic activity. Fe^3+^ released from orthodontic appliances could reduce hemolytic activity [17]. Many environmental factors, such as extracellular pH and the concentrations of alkali metal cations can affect the virulence of *Candida* isolates. Generally speaking, *Candida* species can grow at relatively high NaCl concentrations [25], although salt has a negative impact on some virulence characteristics [26]. A relationship exists between the formation of *C. albicans* hyphae and the intracellular concentration of potassium [27]. Yeast species have transport systems to maintain homeostasis in the presence of a high ratio of potassium and sodium concentrations [28, 29]. Most yeasts have only one type of antiporter to efficiently transport both potassium and sodium cations from the cells [29, 30]. The Na^+^/H^+^ antiporter is an important transport system whose activity can influence the tolerance of *Candida* strains to high external concentrations of alkali metal cations [31].

A previous study showed that Ca^2+^ can inhibit the bactericidal effect of human lactoferrin [32]. Although the reason for this inhibitory effect on candidacidal activity remains unclear, it could be a result of many factors [33]. Researchers previously reported the extracellular cation concentration influenced the bactericidal effect of lactoferrin [34] and assumed that human lactoferrin interacted with the *C. albicans* cytoplasmic membrane in a manner that made it candidacidal [35]. More detailed studies investigating the mechanisms for the decreased hemolytic activity observed in the presence of electrolytes are required.

**Fig. 4:**
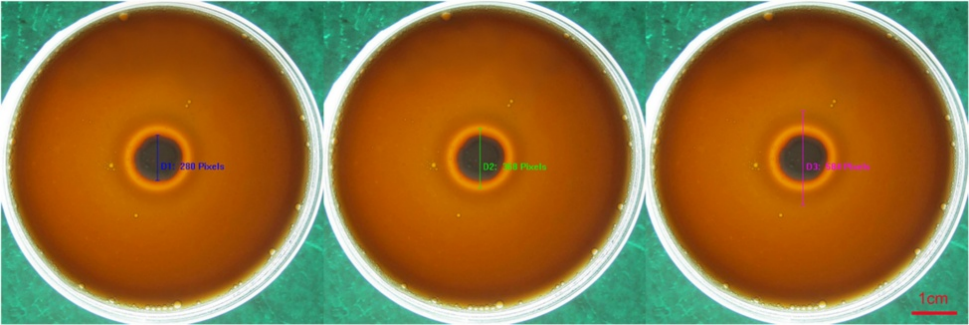
Two different types of hemolytic activity observed around the yeast. The terms hemolytic index (Hi = D2/D1) and peripheral hemolytic index (Hp = D3/D1) were used to indicate these two different forms of hemolysis. The first term denotes a highly translucent ring and the second term describes a peripheral dark halo

In this study, we tested the hemolytic activity of 25 *Candida* isolates. At 48 h post-inoculation, two different types of hemolysis were observed surrounding the yeast (Fig. 4). The first type produced a highly translucent outer ring, and the second type produced a dark peripheral halo, hence, the terms hemolytic index and peripheral hemolytic index were used to describe these two types of hemolysis, respectively. The results conclusively indicated that there was a strong correlation between Hi and Hp. Future work may include an in-depth study of *Candida* hemolysis using chemical analysis to determine the differences between peripheral hemolysis and translucent hemolysis by investigating their chemical composition, properties, structure, and functions.

## Conclusions

Our clinically relevant findings indicate that different electrolytes can modulate the activity of hemolysins, potential virulence factors found in *Candida* species. All the electrolytes tested in this study produced a decrease in the hemolysis observed by the *Candida* species. To our knowledge, this is the first time that a correlation between the peripheral hemolytic index and the translucent hemolytic index has been identified. The results of this study expand our knowledge of factors affecting *Candida* hemolysis.

## Abbreviations

Saps: Secreted aspartate proteinase
ATCC: American Type Culture Collection
Hi: The hemolytic index
Hp: The peripheral hemolytic index
ANOVA: Analysis of variance
